# Periostin Mediates Oestrogen-Induced Osteogenic Differentiation of Bone Marrow Stromal Cells in Ovariectomised Rats

**DOI:** 10.1155/2020/9405909

**Published:** 2020-04-29

**Authors:** Chunrong Li, Xin Li, Xian Wang, Pei Miao, Jia Liu, Cuixia Li, Doudou Li, Weiwei Zhou, Zuolin Jin, Meng Cao

**Affiliations:** State Key Laboratory of Military Stomatology & National Clinical Research Centre for Oral Diseases & Shaanxi Clinical Research Centre for Oral Diseases, Department of Orthodontics, School of Stomatology, The Fourth Military Medical University, 145 West Changle Road, Xi'an, Shaanxi 710032, China

## Abstract

Osteoporosis is a metabolic disease that results in the progressive loss of bone mass, which, in postmenopausal women, is related to oestrogen deficiency. Periostin (POSTN) plays a key role in the early stages of bone formation. However, whether POSTN participates in oestradiol regulation of osteogenic differentiation of bone marrow stromal cells (BMSCs) from ovariectomised (OVX) rats remains unclear. *In vivo*, using microcomputed tomography (micro-CT), immunohistochemistry, and dynamic analysis of femurs, we found that 17*β*-E2 promotes bone formation and POSTN expression at the endosteal surface. *In vitro*, 17*β*-E2 upregulated POSTN expression in OVX-BMSCs. POSTN overexpression activated the Wnt/*β*-catenin signalling pathway and enhanced osteogenic differentiation of OVX-BMSCs. Furthermore, knockdown of *Postn* blocks the involvement of 17*β*-E2 in the osteogenic differentiation of OVX-BMSCs. Collectively, our study indicated the role of POSTN in the osteogenesis and stemness of OVX-BMSCs and proves that 17*β*-E2 reduces osteoporosis and promotes osteogenesis through the POSTN-Wnt/*β*-catenin pathway. POSTN could, therefore, be a novel target gene for anti-osteoporosis therapies.

## 1. Introduction

Postmenopausal osteoporosis is a systemic skeletal disease characterised by reduced bone mass and deterioration of bone microstructure, resulting in increased bone fragility and susceptibility to fracture [1, 2]. With an aging population, postmenopausal osteoporosis is becoming a major global health problem [3, 4].

Oestrogen is a key hormone in bone remodelling and bone morphology maintenance [5]. Oestrogen deficiency reduces the osteogenic capacity of bone marrow stromal cells (BMSCs) and increases osteoclast formation, causing defects in bone formation and osteoporosis [6]. Previous studies [7] have shown the osteoprotective action of oestrogen by regulating bone formation. However, bones are not the only target structures of oestrogen; long-term use of oestrogen also increases the risk of breast tumours and cardiovascular diseases [8].

Periostin (POSTN), a secreted extracellular matrix (ECM) protein, is mainly expressed in the periosteum and the periodontal tissue and plays a vital role in regulating bone metabolism [9, 10]. Notably, *Postn*-deficient mice can cause low bone mass [11]. In contrast, *Postn* mRNA and POSTN levels are rapidly upregulated by mechanical stimuli and the parathyroid hormone (PTH) [12, 13]. Moreover, POSTN may directly or indirectly stimulate Wnt/*β*-catenin signalling [13], a process that controls bone homeostasis. The canonical Wnt pathway specifically promotes the osteogenesis of murine BMSCs and osteoprogenitor cells through upregulation of osteoblast-related genes [13–15].

Thus, both 17*β*-E2 and POSTN can promote bone formation and may regulate osteogenesis via similar mechanisms; however, to our knowledge, no studies, to date, have investigated the existence of internal links between these two elements during osteogenic differentiation. Therefore, the primary aim of this study was to analyse the role of POSTN in oestrogen-deficiency-related postmenopausal osteoporosis. Our results showed that POSTN might participate in the osteodifferentiation of OVX-BMSCs through the oestrogen-POSTN-Wnt/*β*-catenin pathway. Thus, POSTN could be a novel target gene for anti-oestrogen-deficiency-related osteoporosis.

## 2. Materials and Methods

### 2.1. Animal Studies

Eight-week-old female Sprague Dawley (SD) rats, weighing 200–220 g, were obtained from the Animal Centre of the Fourth Military Medical University (Xi'an, China). The animal welfare and all procedures were performed according to the Guide for the Care and Use of Laboratory Animals and approved by the Ethics Committee of the Fourth Military Medical University. The rats were intraperitoneally injected with 3% pentobarbital sodium (0.2 ml/100 g; Sigma-Aldrich) for general anaesthesia and randomised to undergo sham surgeries or ovariectomies. After 6 weeks, 17*β*-oestradiol (10 *μ*g/kg; Sigma-Aldrich, St. Louis, MO, USA) was injected subcutaneously into the rats in the oestrogen treatment group, once every 3 d. The sham and ovariectomised groups were injected with the same dose of corn oil. At 20 weeks of age, the rats were sacrificed for further analysis.

### 2.2. Microcomputed Tomography Scanning and Analysis

A microcomputed tomography (Micro-CT) scanner (*μ*CT40; Scanco Medical, Brütisellen, Switzerland) was used for live animal scanning, and Micview V 2.1.2 software (Siemens Inveon, Germany) was used for three-dimensional reconstruction. The distal femurs of rats were scanned at 80 kV, 456 *μ*A, 3000 ms exposure time, and a rotation/scan angle of 360°. Three-dimensional images of the 1.5 mm metaphyseal regions were reconstructed. Femoral cortical bone thickness (CtTh), bone mineral density (BMD), and bone volume/total volume (BV/TV) were determined at 8, 14, 16, and 20 weeks.

### 2.3. Dynamic Analysis of Bone Growth

To assess bone growth, 18-week-old rats were injected intraperitoneally with 0.5% calcein (10 mg/kg; Sigma-Aldrich) and 3% xylenol orange (90 mg/kg; Energy Chemical) on days 2 and 8, respectively. After 2 days, the rats were sacrificed, and the bilateral femurs were peeled off and fixed in 4% paraformaldehyde at 4°C for 2 days. After 4 h of flushing, the femurs along the long axis were embedded and sliced at 50 *μ*m thickness with a Leica SP1600 microtome (Leica, Germany). Laser confocal microscopy (FluoView™ FV1000, Olympus Corporation, Japan) was used to observe and photograph the femurs. Mineral apposition rate (MAR, mcm/d), mineralised surface area (MS/BS, %), and bone formation rate (BFR/BS, mcm^3^/mcm^2^/d) were calculated.

### 2.4. Immunohistochemistry

The femur specimens were washed several times with saline to remove unwanted materials, fixed in 4% paraformaldehyde, decalcified in 10% ethylenediaminetetraacetic acid-phosphate buffered saline, dehydrated, embedded in paraffin, and cut into 4 *μ*m thick sections. The sample was incubated with anti-POSTN primary antibody (rabbit IgG, diluted at 1 : 100; ab14041; Abcam Co., Ltd., Tokyo, Japan) at 4°C for 16 h. Biotin-conjugated goat anti-rabbit polyclonal antibody (1/200; SP-9000; Zhongshan Jinqiao, Beijing, China) was used as the secondary antibody.

### 2.5. Isolation and Culture of BMSCs

Twenty-week-old female rats were sacrificed by injection of excessive 3% pentobarbital sodium (0.4 ml/100 g; Sigma-Aldrich), after which they were sterilized by soaking in 75% alcohol for 30 min and then rinsed. The tibias and femurs of the rats were collected for isolating BMSCs. The bone marrow cavities were repeatedly flushed with minimum essential medium (*α*-MEM) (Gibco, Rockville, MD, USA) containing 100 U/ml penicillin, 100 mg/ml streptomycin (Sigma-Aldrich), and 10% fetal bovine serum (Gibco, USA) in 25 cm^2^ flasks by using a syringe. The bone marrow was incubated at 37°C in 95% humidified air containing 5% CO_2_. Once the BMSCs reached 80% confluency, BMSCs were resuspended at a 1 : 3 dilution. The third passage of BMSCs was used for further cell-based analyses. The cells were seeded at a concentration of 2 × 10^5^ cells per well into 6-well plates. The adherent OVX-BMSCs were treated with 10^−9^ mol/l 17*β*-E2 for 48 h.

### 2.6. Immunofluorescence Analysis

BMSCs from the sham, OVX, and OVX+E2 groups were seeded at a concentration of 2 × 10^5^ cells per well into 6-well plates. After 48 h, the cells were treated as previously described [16] and then incubated with anti-POSTN antibody (ab14041, 1/1000 dilution) overnight at 4°C and with the secondary antibody (green; Alexa Fluor® 488 goat anti-rabbit IgG (H+L); ab150077, 1/1000 dilution) for 1 h. DAPI (4′,6-diamidino-2-phenylindole; 1.43 *μ*M) was used to stain the cell nuclei (blue). Colocalization of POSTN was evaluated using confocal microscopy (OLS4500, Olympus Corporation, Tokyo, Japan). The integrated optical density (IOD) was analysed, and the average positive intensity was calculated using Image-Pro Plus 6.0.

### 2.7. Lentivirus Transduction

When the sham- and OVX-BMSCs reached 60% confluency, BMSCs were stably transfected with a lentivirus carrying the GFP (Lev-GFP) and POSTN-GFP (Lev-POSTN) genes at a multiplicity of infection (MOI) for 48 to 72 h. Then, infection efficiency was assessed.

### 2.8. Real-Time Reverse Transcription-Polymerase Chain Reaction (RT-PCR)

Total RNA was isolated from the sham, OVX, and OVX+E2-BMSCs using the TRIzol® Reagent (Invitrogen, Carlsbad, CA, USA). The cDNA was synthesized from 1 *μ*g total RNA from the cartilage using a SYBR® Premix Ex Tag TM II kit (TaKaRa Bio Inc., Otsu, Shiga, Japan) for RT-PCR analysis. The primer sequences are provided in Table 1.

### 2.9. Western Blot

Total BMSCs proteins were extracted as previously described [16]. Antibodies included anti-POSTN (1 : 800, ab14041), anti-Wnt3a (1 : 5000, ab28472), anti-*β*-catenin (1 : 3000, ab32572), and anti-GAPDH as the housekeeping gene (1 : 5000, ab181602) and secondary antibodies (horseradish peroxidase, 1 : 10000, ab181658), all purchased from Abcam Co., Ltd.

### 2.10. Osteoblast Functional Assay

The third passage of BMSCs (2 × 10^5^ cells/well) was cultured in 6-well plates for 21 days and stained using a 2% Alizarin Red S staining kit (Solarbio, Beijing, China) to observe calcified nodule formation; 2% cetylpyridinium chloride was used to dissolve Alizarin Red-stained nodules for quantitative analysis. The absorbance values were obtained at 540 nm using Biotek Synergy HTX (Biotek, Winooski, VT, USA).

### 2.11. Postn-siRNA Treatment

siRNAs used in this study were synthesized and labelled with FAM carboxyfluorescein, a fluorescein derivative (Shanghai GenePharma, China). The Postn-siRNA duplex sequences were as follows: sense 5′-GCAGUCUUCAGCCUAUUAUTT-3′ and antisense 5′-AUAAUAGGCUGAAGACUGCTT-3′. The negative control siRNA duplex sequences were as follows: sense 5′-UUCUCCGAACGUGUCACGUTT-3′ and antisense 5′-ACGUGACACGUUCGGAGAATT-3′. Prior to gene transfection, OVX-BMSCs were seeded in 6-well plates (2 × 10^5^ cells/well) and treated with 10^−9^ mol/l 17*β*-E2. The cells were then transfected with siRNA duplexes using Lipofectamine 2000 (Lipofectamine™2000, Invitrogen, Carlsbad, CA, USA) in a culture medium that was both serum- and antibiotic-free for 6 h, followed by incubation in regular medium for 48 to 72 h.

### 2.12. Statistical Analysis

Data are presented as the mean ± standard deviation (SD). One-way analysis of variance (ANOVA) was used for determining the differences between the groups. A *P* value of <0.05 was considered significant. All analyses were performed using Prism (6.0; GraphPad Software Inc., La Jolla, CA, USA).

## 3. Results

### 3.1. Oestrogen Promotes Bone Mineralisation, and Maintains Bone Formation

**Figure 1:**
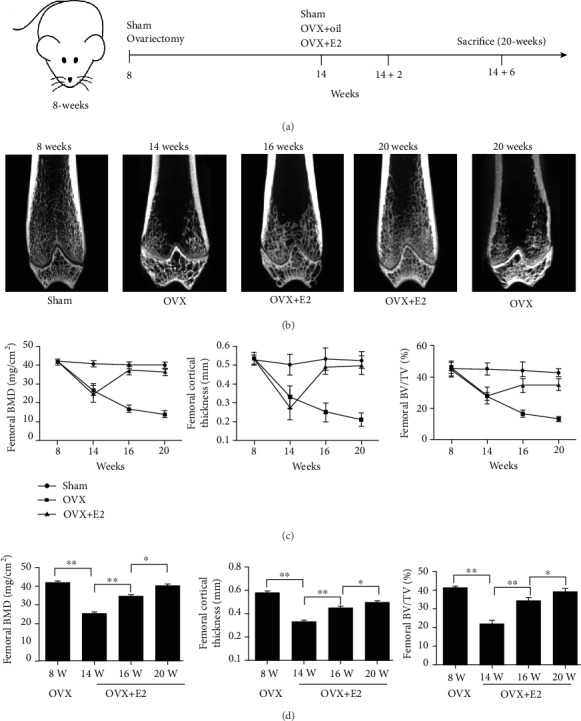
Effect of 17*β*-oestradiol (17*β*-E2) on bones in ovariectomised rats. (a) Timeline of the experiment. (b) Three-dimensional computed tomography images of the distal femurs from representative rats of different ages and with different interventions. (c) BV/TV, CtTh, and BMD of the distal femur of 16- and 20-week-old rats determined via micro-CT. (d) Longitudinal BV/TV, CtTh, and BMD of the distal femur of OVX-rats determined via micro-CT (*n* = 6/group). Bars represent the means ± SD. ^∗^*P* < 0.05, ^∗∗^*P* < 0.01. BV/TV: bone volume fraction; CtTh: cortical thickness; BMD: bone mineral density.

Figure 1(a) shows the timeline of the experiment, treatment, and sacrificial age for the femoral growth study. To elucidate the effect of 17*β*-E2 on cortical and cancellous bones in OVX rats, the femoral bones of the rats were examined using micro-CT (Figure 1(b)). BMD was significantly lower in the OVX than in the sham group at the femur (20 ± 4.583 vs. 41 ± 3 mg/cm^2^, respectively, *P* < 0.001) at 14 weeks of age. Femoral CtTh was also lower in the OVX group than in the sham group at 14 weeks of age (Figure 1(c)), and the 17*β*-E2-stimulated femoral BM increased over 2 weeks. We found that 17*β*-E2 significantly increased CtTh and BV/TV at 16 weeks. BMD, CtTh, and BV/TV at the femur were slightly higher in the OVX+E2 group at 20 weeks than in the OVX group at 16 weeks (Figure 1(d)).

**Figure 2:**
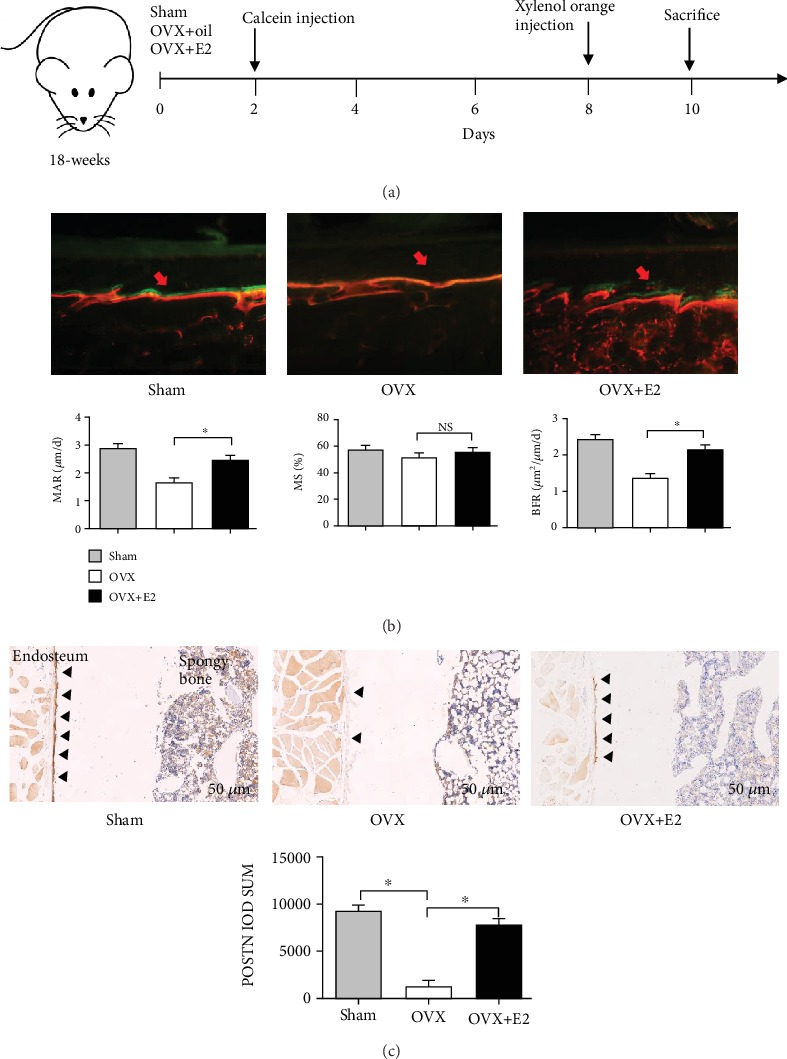
Bone formation and POSTN expression are increased by 17*β*-oestradiol (17*β*-E2) in the endosteum of ovariectomised rats. (a) Timeline of the experiment. (b) MAR, MS, and BFR, as determined via calcein (green) and xylenol orange (red) staining, are shown in the photomicrographs (scale bar: 20 *μ*m) in longitudinal undecalcified sections of femurs from 18-week-old female rats. (c) Immunohistochemical staining of POSTN in longitudinal sections of the proximal femur of the rats 6 weeks after 17*β*-E2 treatment shows increased POSTN expression at the endosteum (Es). Magnification: ×10. In the photomicrographs, POSTN is indicated by the arrows (*n* = 6/group). Bars represent the means ± SD. ^∗^*P* < 0.05. NS: not significant; MAR: mineral apposition rate; MS/BS: mineralised surface area; BFR: bone formation rate.

Calcein and xylenol orange were injected to measure the dynamic bone formation of OVX+E2 rats (Figure 2(a)). In 18-week-old OVX+E2 rats, compared with their OVX littermates, bone formation was also accelerated in the distal femur, which exhibited a 38.8% increase in MAR with no change in mineralising surface, resulting in an overall 30% increase in BFR at the endosteal surface (Figure 2(b)).

### 3.2. POSTN Expression Is Promoted by 17*β*-E2 In Vivo

We next ascertained whether 17*β*-E2-stimulated POSTN expression might play an additional role in bone formation. Hence, we performed immunohistochemical staining of POSTN in the distal femur. The results showed that POSTN was more highly expressed in the endosteum of the OVX+E2 group than in the OVX group (Figure 2(c)).

### 3.3. POSTN Expression and Osteogenic Differentiation Is Promoted by 17*β*-E2 In Vitro

**Figure 3:**
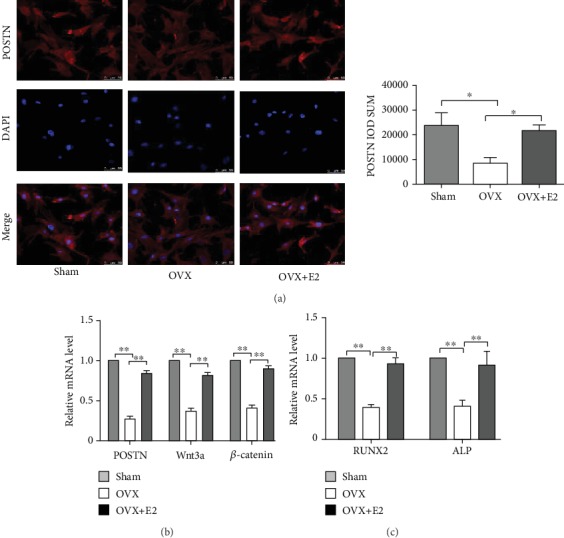
POSTN protein expression in response to 17*β*-oestradiol (17*β*-E2) was increased *in vitro*. (a) Immunofluorescence of POSTN (red) localization in BMSCs. Magnification: ×40. POSTN IOD quantification using Image-Pro Plus 6.0. (b) *Postn*, *Wnt3a*, and *β-catenin* mRNA levels shown, as measured via RT-PCR analysis after 48 h of 17*β*-E2 treatment. (c) *RUNX2* and *ALP* mRNA expressions observed in OVX+E2-BMSCs are higher than in OVX-BMSCs. Bars represent the means ± SD; *n* = 3, ^∗^*P* < 0.05, ^∗∗^*P* < 0.01. BMSCs: bone marrow-derived mesenchymal stem cells; IOD: integrated optical density.

To confirm and further understand the in vivo observations, we investigated the effects of 17*β*-E2 on POSTN, Wnt/*β*-catenin signalling, and bone formation in BMSCs. The images of immunofluorescence revealed that POSTN exhibited higher density in BMSCs from the OVX+E2 than OVX rats (Figure 3(a)) and primarily localized in the cytoplasm of BMSCs (*P* < 0.05). Similar results were observed in the mRNA level of POSTN, as shown in Figure 3(b); meanwhile, 17*β*-E2 significantly increased the level of Wnt3a and *β*-catenin, compared with the OVX group (*P* < 0.01). Moreover, after the induction of osteogenesis, RUNX2 and ALP expression significantly increased in the OVX+E2 than in the OVX group (Figure 3(c)). Collectively, these results suggest that 17*β*-E2 promotes POSTN and activates the Wnt/*β*-catenin signalling pathway.

### 3.4. Overexpression of POSTN in OVX-BMSCs Activates the Wnt/*β*-catenin Signalling Pathway

**Figure 4:**
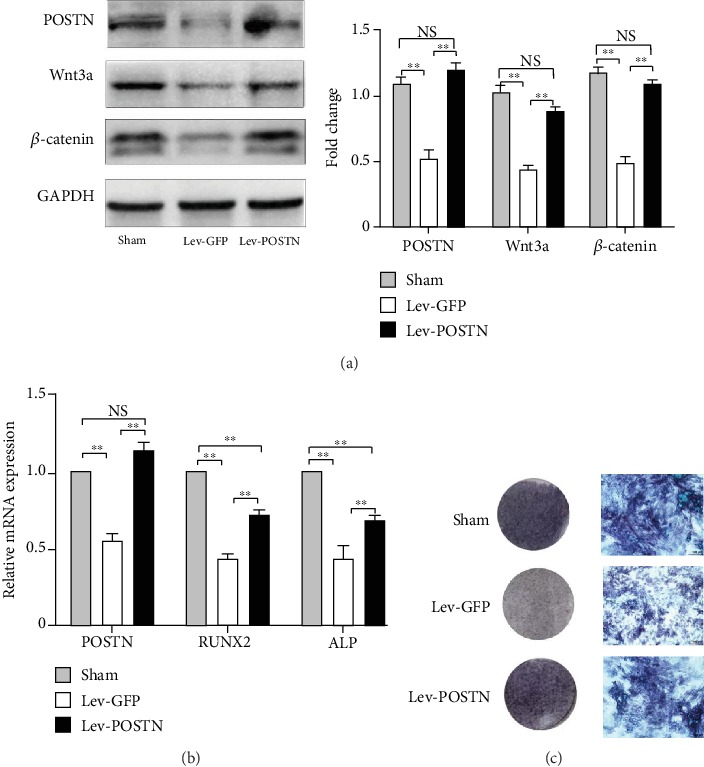
Overexpression of POSTN in OVX-BMSCs increased the expression of POSTN, Wnt3a, *β*-catenin, and osteogenic differentiation. (a) Higher expression of POSTN, Wnt3a, and *β*-catenin was observed via western blot in the Lev-POSTN than in the Lev-GFP group; there was no significant difference relative to the sham group. (b) RT-PCR showing partly restored mRNA levels of *Postn*, *RUNX2*, and *ALP* of OVX-BMSCs following POSTN overexpression. (c) Overexpression of POSTN partially restored ALP activity of OVX-BMSCs. Bars represent the mean ± SD; *n* = 3, ^∗∗^*P* < 0.01. NS: not significant.

As we observed a 17*β*-E2-mediated activation of the Wnt/*β*-catenin signalling pathway and an increase in osteogenic differentiation in oestrogen-deficient BMSCs, we assessed whether POSTN played a similar role in the OVX-BMSCs. Lentiviruses expressing POSTN-GFP were produced to overexpress POSTN; transfection with lentivirus carrying the GFP (Lev-GFP) was regarded as the control. The expression of POSTN, Wnt3a, and *β*-catenin was higher in the Lev-POSTN group than in the Lev-GFP group, while the expression represented no significant difference between the Lev-POSTN group and the sham-BMSCs group (Figure 4(a)). To investigate the osteoblastic differentiation, RT-PCR and ALP staining were performed 7 days after osteogenic induction. The results showed that the overexpression of POSTN partly reversed *RUNX2* and *ALP* levels (Figure 4(b)) and partially restored the ALP activity of OVX-BMSCs (Figure 4(c)). In short, our results suggest that POSTN is an important osteoblast-specific factor that can reduce the dysfunction of OVX-BMSCs and partly restore the function closer to that of BMSCs.

### 3.5. POSTN Mediates 17*β*-E2 Participation in the Osteogenic Differentiation of OVX-BMSCs via the Canonical Wnt Pathway

**Figure 5:**
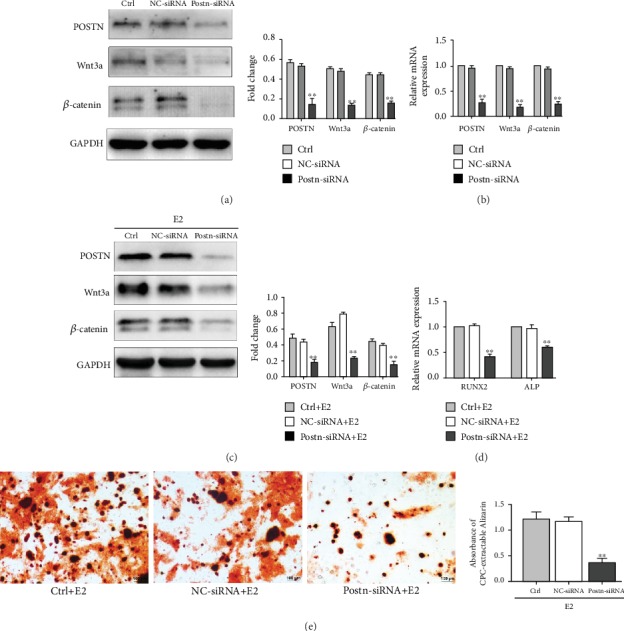
Inhibition of the expression of POSTN, Wnt3a, and *β*-catenin and osteogenic differentiation in OVX-BMSCs with or without 17*β*-E2 using *Postn*-siRNA. (a) Expression of POSTN, Wnt3a, and *β*-catenin was lower in OVX-BMSCs with *Postn*-siRNA compared with the negative control siRNA and control groups. (b) The mRNA levels of POSTN, Wnt3a, and *β*-catenin were lower in OVX-BMSCs with *Postn*-siRNA compared with the negative control siRNA and control groups. (c) Treatment of OVX-BMSCs with 10^−9^ M 17*β*-E2 did not reverse the levels of POSTN, Wnt3a, and *β*-catenin in the *Postn*-siRNA group. (d) The mRNA levels of *RUNX2* and *ALP* in OVX+E2-BMSCs with *Postn*-siRNA were lower than those in the negative control siRNA and control groups. (e) *Postn* knockdown significantly decreased the mineralised node formation. Bars represent the means ± SD; *n* = 3, ^∗∗^*P* < 0.01.

To investigate the role of POSTN in oestrogen regulation of bone formation, OVX-BMSCs were treated with *Postn*-siRNA and negative control siRNA. The transfection efficiency of siRNA was detected, and POSTN, Wnt3a, and *β*-catenin levels were determined via western blot and RT-PCR. POSTN protein and mRNA expression decreased by 70.15 ± 2.2% and 73.33 ± 2.08%, respectively, following BMSCs treatment for 24 h with *Postn*-siRNA (*P* < 0.001, vs. control; Figures 5(a) and 5(b)).

To further evaluate the relationship between POSTN and the Wnt/*β*-catenin signalling pathway under 17*β*-E2 stimulation, the OVX-BMSCs were transfected with siRNA for 2 h prior to treatment with 17*β*-E2. POSTN, Wnt3a, and *β*-catenin were measured by western blotting. Our data showed that POSTN, Wnt3a, and *β*-catenin protein expression was lower in the *Postn*-siRNA group than in the negative control siRNA group (Figure 5(c)).

Recently, we demonstrated that bone loss during osteoporosis was partly due to the decline in BMSCs osteogenic differentiation [17]. To investigate whether the differentiation defect in OVX-BMSCs is associated with the decline in POSTN, we induced osteogenic differentiation in OVX-BMSCs and added 17*β*-E2 (10^−9^ M) in the osteogenic medium. Seven days after osteogenic induction, the mRNA levels of the osteogenic markers, *ALP* and *RUNX2*, were significantly lower than those in the negative control siRNA group (Figure 5(d)).

In addition, Alizarin Red S staining showed that compared with the control and negative control siRNA groups, *Postn*-siRNA had a negative effect on calcium deposition after 21 days of osteogenic induction. The positive rate of standardized region staining for Alizarin Red S in the *Postn*-siRNA group was 30.8 ± 2.3%, which was significantly lower than that in the negative control siRNA group (Figure 5(e)).

## 4. Discussion

In postmenopausal osteoporosis, the key to treatment is to restore damaged bone formation ability and improve metabolic and functional remodelling [18]. Since oestrogen is the main hormone regulator of bone metabolism, considerable interest exists for unravelling the potential pathways of oestrogen in bone protection. These observations are in accordance with previous data indicating that oestrogen treatment via oestrogen pellet implantation (OVX+E2) for 2 weeks in oestrogen receptor ERa+/+ OVX mice elicited a significant increase in femur bone mass [7]. Previous results have shown that oestrogen regulates osteogenic differentiation of BMSCs via the ER*β*-SATB2 pathway to prevent osteoporosis [19]. It was also reported that chloride channels are activated by oestrogen binding to ER*α* in the cell membranes of MC3T3-E1 osteoblasts [20]. However, possible mechanisms for 17*β*-E2 effects on bone formation were less clearly understood. Here, we demonstrated the active role of POSTN in 17*β*-E2-mediated osteogenic differentiation, revealing POSTN as a candidate target for the prevention and treatment of osteoporosis.

A relationship between POSTN and 17*β*-E2 was first suggested by the observation that oestrogen regulates Postn gene expression and osteoblastic differentiation in human periodontal ligament cells via ER*β* [21]. POSTN could be detected along the femoral endosteum in the OVX+E2 group but not in the OVX group (Figure 2(b)). This may largely be explained by the role of oestrogen in limiting periosteal bone expansion but stimulating endosteal bone apposition. A further complexity regarding the effects of oestrogen on bone formation is that these effects appear to be bone envelope-specific [22]. In contrast, POSTN in longitudinal sections of the proximal tibia of Postn+/+ mice after PTH treatment exhibited increased POSTN expression at the periosteum but not at the endocortical surfaces [13]. It is likely, therefore, that POSTN plays an important role in bone formation and morphologic maintenance. The polarized expression of POSTN may also have implications regarding the differential effects and potency of PTH and 17*β*-E2 at the periosteal vs. endocortical surfaces. In vitro experiments also demonstrated that 17*β*-E2 promotes POSTN expression of OVX-BMSCs, with POSTN being localized in the cytoplasm as shown by immunofluorescence analyses. These observations are consistent with previous data indicating that POSTN is primarily located in the cytoplasm of wild-type and CTLA4-modified BMSCs [23]. There are two main mechanisms that may promote POSTN activation in OVX-BMSCs. Firstly, 17*β*-E2 may bind to ER*α* or ER*β* in the cell membranes of OVX-BMSCs in a ligand-dependent way to modulate transcription and promote POSTN activation. Secondly, 17*β*-E2 may promote POSTN expression through cytokines. Oestrogen increases the release of transforming growth factor beta (TGF-*β*), a powerful stimulator of earlier phases of differentiation and osteoblastic recruitment, and TGF-*β* can also induce POSTN secretion from BMSCs to support bone formation [9].

The Wnt/*β*-catenin pathway plays an important regulatory role in osteogenic differentiation and has become a hot spot for research in bone disease in recent years [24, 25]. Previous studies have shown that POSTN may regulate Wnt signalling, a crucial mediator of bone anabolism, via autocrine/paracrine mechanisms [13]. In this study, we demonstrated that POSTN is targeted by oestrogen and plays an important role in oestrogen-regulated bone formation processes via the Wnt/*β*-catenin pathway in OVX-BMSCs. *Postn* knockdown by siRNA affected the differentiation and mineralisation processes induced by 17*β*-E2 in OVX-BMSCs. In addition, the results of this study showed that Wnt pathway activity and calcium deposition were also inhibited by *Postn*-siRNA. In comparison, previous studies have shown that POSTN is necessary for the bone anabolic response to mechanical stimuli [26–28]. Notably, POSTN was shown to directly inhibit Sost expression through its integrin *α*V*β*3 receptor, thereby activating the Wnt signalling pathway [29]. Additionally, POSTN has been confirmed to indirectly reduce *β*-catenin degradation, by blocking PTEN, which activates *β*-catenin degradation. The early stage of osteoblast differentiation involves cellular proliferation. During this phase, the expression of RUNX2, a key osteoblastic transcription factor [30], essential for osteogenic differentiation, is established in BMSCs. Upon Wnt signalling, *β*-catenin nuclear translocation induces RUNX2 expression [31] via the direct binding of TCF/LCF cotranscription factors [32]. However, the complex network of signalling pathways and the potential involvement of POSTN to directly stimulate Wnt/*β*-catenin signalling still require further exploration. Meanwhile, ovariectomised rats should be treated with Lev-OVX-POSTN BMSCs to verify the effectiveness of this therapy in oestrogen-deficiency-related osteoporosis.

In conclusion, our current findings have demonstrated that 17*β*-E2 can modulate the early stages of bone formation in vivo, stimulate the expression of POSTN, and enhance Wnt/*β*-catenin pathway activity in OVX-BMSCs. Taken together, these results suggest that POSTN may be targeted by 17*β*-E2 to regulate osteogenic differentiation via the Wnt pathway. In turn, this novel network between POSTN and osteogenic induction during degenerative bone disease may provide novel targets and strategies for the development of alternative therapies and biomarkers for postmenopausal osteoporosis.

## Figures and Tables

**Table 1:** Primers used for RT-PCR.

Primer name	Sense (5′ to 3′)	Antisense (5′ to 3′)
GAPDH	GTTACCAGGGCTGCCTTCTC	GGGTTTCCCGTTGATGACC
*Postn*	TGCAAAAAGACACACCTGCAA	CCGAAGTCAATGGGGCTCTT
*RUNX2*	CGCCTCACAAACAACCACAG	CGCCTCACAAACAACCACAG
*ALP*	GCCCAGTGCCTTAAACGTGA	CCAGGCTTCTTCACTGGTCC

*Postn:* periostin.

## Data Availability

The data used to support the findings of this study are included within the article.
